# Silver Halides as Strategic Functional Materials: Resource Potential and Technological Evolution (1975–2025)

**DOI:** 10.3390/ma19122636

**Published:** 2026-06-18

**Authors:** Medet Junussov, Zamzagul T. Umarbekova, Maxat K. Kembayev, Ravil R. Gadeev, Gulnur Mekenbek, Moldir A. Mashrapova

**Affiliations:** 1 School of Mining and Geosciences, Nazarbayev University, Kabanbay Batyr Street 53, 010000 Astana, Kazakhstan; 2 Institute of Geological Sciences Named After K.I. Satpayev, Kabanbay Batyr Street 69, 050010 Almaty, Kazakhstan; 3 Institute of Geology and Oil-Gas Business, Satbayev University, Satbayev Street 22, 050013 Almaty, Kazakhstan

**Keywords:** silver, silver halides, bibliometrics, Web of Science, VOSviewer, photocatalysis, functional materials

## Abstract

**Highlights:**

**What are the main findings?**

- Global silver halides research spans natural and synthetic materials.
- Publication output surged post-2005, driven by nanotechnology and photocatalysis.
- Six thematic clusters identified: environment, catalysis, crystal growth, structure, interface, electroanalysis.

**What are the implications of the main findings?**

- Silver halides are key multifunctional materials for next-generation technologies.
- Nanostructured and hybrid designs enable advanced photocatalysis and optical devices.
- Insights guide sustainable synthesis, AI-assisted design, and interdisciplinary research.

**Abstract:**

Driven by advances in multifunctional materials design, silver halides—both natural (AgCl, AgBr, AgI, and mixed phases such as embolite) and synthetic—have emerged as versatile functional materials characterized by tunable crystallography, phase stability, and compositional variability. This study investigates global research trends, interdisciplinary development, and emerging application areas of silver halides through a bibliometric analysis of 23,841 publications indexed in the Web of Science (1975–2025). CDPI, TELM, VOSviewer, and Excel were employed to evaluate publication growth, disciplinary integration, and thematic evolution. Research output increased markedly after 2005, reaching approximately 700–1000 publications annually during 2020–2025. China (18.3%) and the United States (17.5%) were the leading contributors, while the Chinese Academy of Sciences, Russian Academy of Sciences, and CNRS showed the highest scientific impact. Materials Science Multidisciplinary (CDPI = 0.72), Chemistry Multidisciplinary (0.70), and Physical Chemistry (0.67) exhibited the strongest interdisciplinary integration, whereas Nanoscience and Nanotechnology demonstrated the fastest growth. Keyword co-occurrence analysis identified six major research domains focused on functional materials engineering, including environmental remediation, catalysis, crystal growth, antibacterial materials, interfacial processes, and electroanalytical systems. Recent studies increasingly emphasize structure–property relationships and synthetic control of crystal size, morphology, and surface characteristics to enhance performance in photocatalysis, sensing, antimicrobial coatings, and advanced optical applications. Overall, the results highlight the growing importance of silver halides as strategic functional materials and provide a quantitative framework for future research and technological development. A limitation of this study is its exclusive reliance on the Web of Science database, which may underrepresent relevant publications indexed elsewhere.

## 1. Introduction

Silver halides—specifically silver chloride (AgCl), silver bromide (AgBr), silver iodide (AgI), and, to a lesser extent, silver fluoride (AgF)—are light-sensitive inorganic compounds with broad industrial relevance and notable economic significance.

**Figure 1:**
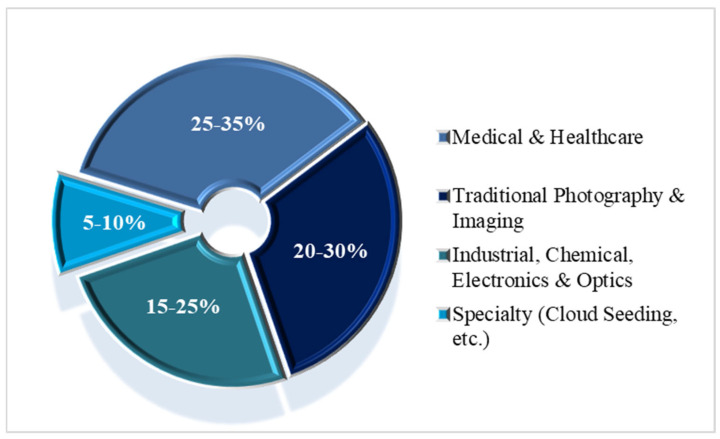
Illustration of the major utilization sectors of silver halides (AgCl, AgBr, AgI, and AgF), with values compiled from literature sources [1,2,3,4,5,6,7,8,9]. Percentages are estimated from normalized aggregation of reported application frequencies across reviewed studies, grouped into several categories as shown.

The growing demand for advanced technologies has highlighted the strategic importance of silver halides across multiple sectors. These compounds are employed in photographic and imaging technologies, sensor and detection systems, medical and healthcare applications, industrial and chemical processes, and electronic and optical devices, as well as specialized applications such as weather modification. Historically, the photographic sector was the primary consumer of silver halides, accounting for approximately 40% of global silver consumption in the late twentieth century. In recent decades, the rise of sensor technologies—including optical, plasmonic, and photocatalytic sensors—has driven increasing research and application, reflecting the evolving multifunctionality of silver halides phases. Following the advent of digital imaging, current utilization within photographic and imaging applications has decreased to an estimated 20–30%, encompassing medical X-ray films, specialty photographic papers, and high-resolution imaging systems [1,2,3], see Figure 1. The silver halides photographic paper market remains economically significant, with an estimated valuation of USD 1.2 billion, sustained by applications in medical diagnostics and artistic photography [3].

The medical and healthcare sector currently accounts for approximately 25–35% of silver halides utilization. This demand is driven primarily by the antimicrobial properties of AgCl and nanoscale silver halides materials, which are employed in wound dressings, coatings for medical devices, diagnostic electrodes (e.g., Ag/AgCl sensors), and water purification systems [4,5,6]. Beyond antimicrobial applications, silver halides are also integral to electrochemical sensing platforms and optical devices, such as photochromic lenses, where embedded silver halides microcrystals undergo reversible photochemical transformations in response to ultraviolet irradiation [5,7]. Industrial and chemical applications constitute approximately 15–25% of total silver halides consumption, encompassing their use in analytical chemistry, catalysis, light-sensitive coatings, and specialized synthetic procedures [8]. Similarly, electronics and optical applications account for 15–25% of utilization, including infrared optical fibers, advanced photodetectors, and other precision optical components [9], see Figure 1.

Specialty applications, particularly cloud seeding utilizing AgI, represent roughly 5–10% of overall consumption. The ice-mimetic hexagonal crystal structure of AgI facilitates nucleation in weather modification programs. The global silver iodide market is projected to expand from USD 339 million in 2024 to USD 570 million by 2032, while the broader cloud seeding sector is anticipated to reach USD 4.14 billion by 2034 [10,11,12]. Additionally, silver halides are increasingly explored in environmental remediation, particularly in visible-light-driven photocatalytic degradation of pollutants, which underscores their growing role in sustainable water purification technologies [5]. Silver fluoride (AgF), in contrast, remains a minor component in industrial applications, largely confined to laboratory fluorination chemistry and limited dental uses [8].

Silver halides can be characterized in terms of (1) natural occurrence and (2) synthetic production, both of which underpin their strategic significance in various technological applications. Silver halides, including AgCl, AgBr, AgI, and AgF occur naturally to differing extents. AgCl is present as the mineral chlorargyrite, AgBr as bromargyrite, and AgI as iodargyrite, forming under arid, oxidizing conditions where halogen-bearing solutions interact with native silver and associated silver minerals within the supergene zones of hydrothermal veins or polymetallic deposits. In contrast, AgF is exceedingly rare and lacks significant mineral occurrences. Given the limited natural availability of silver halides relative to sulfides and sulfosalts, industrial and laboratory synthesis predominates. AgCl, AgBr, and AgI are typically produced by precipitating soluble silver salts, such as silver nitrate, with chloride, bromide, or iodide sources, yielding finely divided, light-sensitive crystals widely applied in photographic emulsions, imaging systems, and optical devices. AgF, however, is synthesized almost exclusively through reactions of silver salts (e.g., silver carbonate or silver oxide) with fluoride sources, producing a highly soluble compound with distinct chemical properties and specialized applications in chemical synthesis and fluorination processes. Collectively, the combination of scarce natural occurrence and controlled synthetic preparation underscores the strategic, technological, and chemical importance of silver halides [4,5,13,14,15].

Growing environmental regulations, together with the application of big data and AI to accelerate materials research, are rapidly transforming the scientific landscape, highlighting the strategic importance of silver halides due to their unique optoelectronic and photochemical properties in applications such as sensors, imaging systems, and environmental technologies. This emphasizes how material innovations are closely intertwined with broader scientific and technological trends.

Bibliometric approaches are widely employed to organize and analyze large volumes of scientific data, utilizing comprehensive databases such as Web of Science, Scopus, and ScienceDirect [16,17,18,19]. These platforms facilitate assessment of research impact, trend analysis, and evaluation of geographic and institutional contributions. Tools such as Bibexcel, NetDraw, CiteSpace, Pajek, TDA, and VOSviewer enable network visualization, co-citation mapping, and detection of emerging trends, supporting comprehensive bibliometric studies that guide research evaluation and strategic planning [17,20,21].

This study presents the first systematic bibliometric review of global silver halides research, covering publications from 1975 to 2025 and based on 23,841 records indexed in the Web of Science database. To provide a robust analytical framework, the study integrates the Cross-Disciplinary Publication Index (CDPI) and the Technology–Economic Linkage Model (TELM), complemented by VOSviewer and Microsoft Excel analyses. This approach enables a comprehensive assessment of publication trends, leading researchers and institutions, keyword co-occurrence, and dominant research themes, identifying key contributors and global innovation networks. The study aims to: (i) analyze global research trends, publication dynamics, and scientific output related to silver halides as strategic functional materials over the period 1975–2025; (ii) identify key knowledge gaps in their resource potential and applications; (iii) assess the evolution of research themes and technological development pathways; (iv) examine their interdisciplinary integration across chemistry, materials science, physics, and environmental sciences; (v) evaluate their role in sustainability and future research directions. By offering the first dedicated bibliometric overview of this field, the study provides quantitative and thematic insights into the evolution of silver halides science, informs policy and industrial strategies for material development, and supports evidence-based decision-making for future innovation and sustainable technological progress.

## 2. Materials and Methods

### 2.1. Data Collection

**Figure 2:**
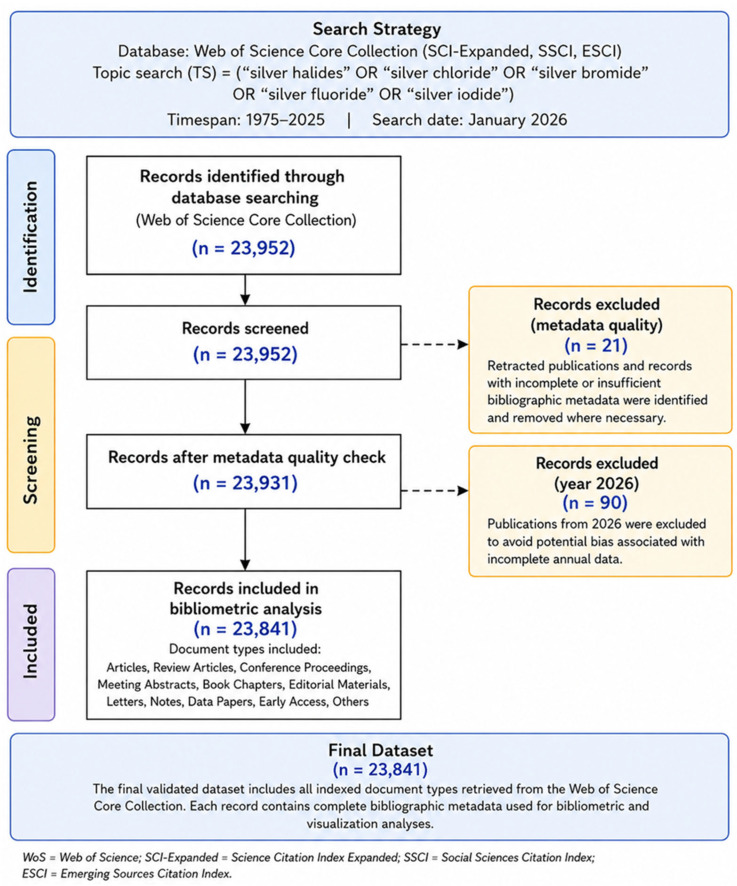
PRISMA-style flow diagram showing the identification, screening, and inclusion of publications retrieved from the Web of Science Core Collection for the bibliometric review.

This study constructed a comprehensive dataset using the Science Citation Index Expanded (SCI-Expanded), Social Sciences Citation Index (SSCI), and Emerging Sources Citation Index (ESCI) within the Web of Science (WoS) Core Collection (Figure 2). An advanced search query was performed using the core terms “silver halides,” “silver chloride”, “silver bromide”, “silver fluoride”, and “silver iodide”, combined with OR operators and applied across all searchable fields.

Topic search (TS) = (“silver halides” OR “silver chloride” OR “silver bromide” OR “silver fluoride” OR “silver iodide”) was used as the TS query in the Web of Science Core Collection (title, abstract, author keywords, and Keywords Plus).

The search covered the publication period from 1975 to 2025. The initial query retrieved 23,952 records representing scientific literature related to silver halides across multiple disciplines. The search was conducted in January 2026 to ensure reproducibility. The selected keywords were identified based on their frequent occurrence in highly cited publications and their interdisciplinary relevance.

To ensure transparency, reproducibility, and methodological rigor consistent with established bibliometric practices, the data collection and filtering procedures were systematically refined. Bibliographic records were retrieved from the WoS Core Collection using standardized topic-based queries similar to those applied in recent large-scale bibliometric studies. Prior to analysis, the dataset underwent several quality-control procedures (Figure 2):

- Duplicate records were checked using WoS export screening and DOI matching. No duplicate records were identified.
- Author names, affiliations, and keywords were standardized to improve consistency.
- Retracted publications and records with incomplete or insufficient bibliographic metadata were identified and removed where necessary. A total of 21 records were excluded during this step.
- Journal indexing consistency was verified within the WoS Core Collection.
- Publications from 2026 were excluded to avoid potential bias associated with incomplete annual data. A total of 90 records published in 2026 were removed.

Following the screening and validation procedures, 23,931 records remained after metadata quality assessment, and the final validated dataset consisted of 23,841 publications.

The final validated dataset consisted of 23,841 publications, including articles, review papers, conference proceedings, meeting abstracts, book chapters, and other. Each record retained complete bibliographic metadata, including authors, publication year, institutional affiliations, keywords, source titles, citation counts, counties, and subject categories.

Additionally, the WoS Core Collection provides high-quality and well-curated bibliographic data, it does not fully capture engineering conference proceedings, patents, regional publications, non-English literature, and industrial reports. Therefore, the present study is limited to peer-reviewed scientific literature indexed in WoS, and complementary databases such as Scopus, Dimensions, and Lens may provide additional coverage in future studies, particularly for applied and technological domains.

### 2.2. Methods

The Web of Science (WoS) platform was selected as the primary data source due to its extensive coverage of peer-reviewed literature and its recognized reliability for bibliometric analyses. Its rigorous indexing and broad subject representation make it well-suited for examining long-term trends and patterns in silver halides-related research output.

To enable a comprehensive evaluation, this study employed two principal bibliometric frameworks. The Cross-Disciplinary Publication Index (*CDPI*) was applied to assess the interdisciplinary reach of silver halides research by quantifying the diversity of cited WoS subject categories represented in the references cited by each publication. The analysis was based on WoS subject categories assigned to cited references, rather than the subject categories of the citing publications, in order to better capture knowledge integration across disciplines. For each publication *i*, the disciplinary diversity index (*DPI*) was computed as follows (1),

(1) $$DPI=-\sum_{j=1}^{N}pij\;In\;\left(pij\right)$$

(2) $${CDPI}_{i}^{norm}=\frac{CDPIi}{In\;(N)}$$

where *N* represents the total number of distinct WoS subject categories identified among the cited references of publication *i*, and *pij* denotes the proportion of cited references belonging to subject category *j* within publication *i.*

To allow comparison across publications with varying disciplinary coverage, the (DPI) was normalized as follows (2). This normalization produces CDPI values ranging from 0 to 1, where higher values indicate greater interdisciplinarity. This normalization facilitates consistent comparison of interdisciplinary diversity across publications with different numbers of cited-reference disciplines. The approach provides a descriptive quantitative measure of disciplinary integration in silver halides research.

Discipline-pair co-occurrence scores were calculated from the frequency with which two WoS subject categories appeared together within the cited-reference set of a publication. These co-occurrence frequencies were normalized to a 0–1 scale, where higher values indicate stronger interdisciplinary linkage between discipline pairs.

Additionally, the Technology–Economic Linkage Model (TELM) was employed to assess the connection between silver halides-related research, technological innovation, and economic development. This model defines weighted linkages among academic publications, patents, and relevant economic indicators. Patent data were obtained from the PATSTAT and Derwent databases, with technological domains classified according to the International Patent Classification (IPC) and Cooperative Patent Classification (CPC) systems. Research and development (R&D) statistics from the Organisation for Economic Co-operation and Development (OECD) and World Bank indicators were used as sources for economic variables. Particular attention was given to low-carbon and sustainable technology classes, specifically Y02 and Y04S. Publication–patent–economic linkages were established through paper–patent citation matching, author–inventor disambiguation, institutional affiliation alignment, and keyword/IPC/CPC mapping.

*TELM* values (*Lₜ_p_,ₖ*) were computed following Equation (3), where linkage intensities were derived from a normalized bipartite network connecting publications, patent classes (IPC/CPC), and economic indicators.

*TELM* values were computed as follows (3),

(3) $$TELM=\sum_{t=1}^{T}\sum_{P=1}^{P}Wtp\cdot Ltp,k$$

where *Ltp,k* represents the linkage intensity between technology class *t* (as defined by IPC/CPC classifications) and economic indicator *p* (e.g., patent output or R&D activity) for a given region or sector *k*, and *Wtp* denotes weighting factors accounting for citation frequency and network connectivity.

All variables were normalized prior to analysis using z-score normalization to ensure comparability across indicators and reduce scale bias in cross-sector linkage assessment. These linkages were established through multiple channels, including paper–patent citation relationships, assignee–affiliation matches, co-authorship networks, and shared technology classifications, with particular emphasis on patents related to sustainable and low-carbon technologies.

The results obtained from the CDPI and TELM analyses were further validated using statistical approaches. These included comparison with alternative diversity metrics (such as the Herfindahl–Hirschman Index), Pearson and Spearman correlation tests, sensitivity analysis across 5- and 10-year time windows, and robustness checks under different network thresholds.

All statistical tests were reported in the Results section to ensure consistency between TELM formulation and empirical outputs, including correlation matrices between publication intensity, patent activity, and economic indicators.

The Web of Science (WoS) database provides a reliable and consistent foundation for bibliometric analyses because of its coverage of high-impact journals, robust citation tracking system, and stringent journal selection criteria [22]. Its extensive temporal coverage, extending back to the 1960s, enables detailed evaluation of historical research trends and citation dynamics [23,24]. In addition, WoS data are fully compatible with bibliometric visualization software such as VOSviewer, which facilitates the construction and interpretation of complex bibliometric networks [25].

In this study, bibliographic records were retrieved from WoS and processed using Microsoft Excel 2019 for data organization and preliminary management. Bibliometric network construction and visualization were performed using VOSviewer (version 1.6.20), developed by [25]. The software was applied to generate co-authorship, co-citation, bibliographic coupling, institutional collaboration, and keyword co-occurrence networks.

The analysis employed the full counting method, association strength normalization, and VOS clustering based on modularity optimization. Minimum thresholds for authors, documents, and keywords were applied according to dataset size to improve statistical reliability and reduce network noise. Link strength was defined according to network type, representing co-citation frequency, collaboration intensity, or keyword co-occurrence relationships. Cluster numbers were determined automatically by the software rather than being predefined, allowing the network structure to reflect the inherent thematic complexity of the dataset. Sensitivity analyses further confirmed the stability and interpretability of the resulting cluster structures within the intellectual landscape of silver halides research.

Artificial intelligence tools were used only for limited language editing support. All bibliometric data extraction, filtering, calculations, visualization procedures, and network analyses were independently conducted using WoS, Excel, and VOSviewer. AI tools were not used for data interpretation or for generating scientific conclusions.

## 3. Results

### 3.1. Document Types and Annual Distribution of Silver Halides-Related Publications

**Figure 3:**
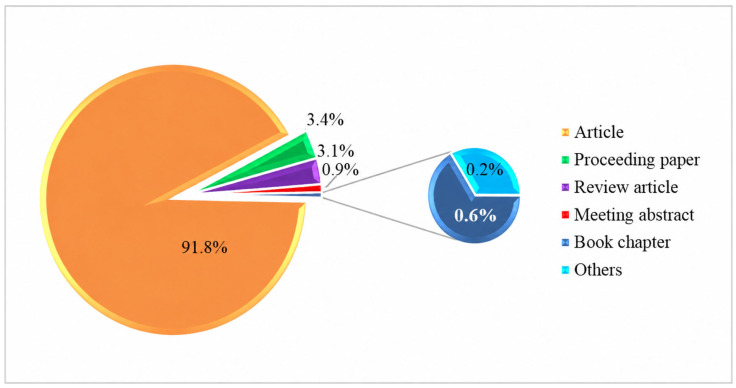
Distribution of silver halides–related publications according to document type based on the WoS Core Collection (1975–2025).

The pie chart in Figure 3 presents the distribution of 23,841 silver halides-related publications indexed in the Web of Science database from 1975 to 2025, categorized by document type. Articles dominate the publication landscape, accounting for 91.8% of all records, indicating that original research constitutes the primary mode of scientific communication in the field of silver halides. Proceeding Papers represent 3.4% of the total, reflecting the role of conference contributions in disseminating emerging research and preliminary findings. Review Articles comprise 3.1%, providing critical syntheses of existing studies and guiding subsequent investigations. Meeting Abstracts account for 0.9%, summarizing research presented at conferences or workshops, while Book Chapters and the “Others” category are minimal, at 0.6% and 0.2%, respectively, encompassing specialized contributions such as edited-volume chapters or non-standard publications. The distribution highlights a strong emphasis on original research articles in silver halides studies, with comparatively limited output in review literature, conference summaries, and other publication types. This pattern underscores the field’s focus on the generation and formal dissemination of new experimental and theoretical findings.

**Figure 4:**
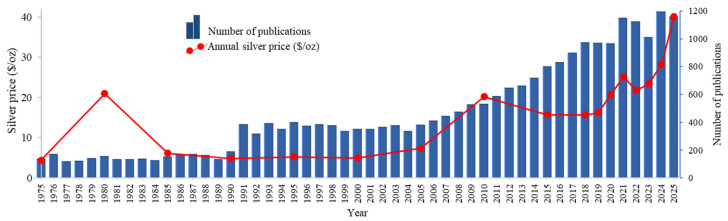
TELM chart showing annual trends in publication output related to silver halides and the corresponding annual silver price between 1975 and 2025 (with silver halides prices approximated by global silver prices).

The TELM chart in Figure 4 illustrates the relationship between annual silver prices (USD per ounce) and the number of scientific publications related to silver halides, and TELM-derived linkage intensities between publication clusters, patent activity (IPC/CPC classes), and economic indicators between 1975 and 2025. Network construction parameters include predefined node inclusion thresholds for publication and patent occurrences, edge formation based on co-occurrence/link strength thresholds, and exclusion of isolated nodes below the minimum threshold. Link strength is defined as the normalized co-occurrence frequency between publication, patent, and economic entities. The economic significance of silver halides is closely associated with the global price of silver, which increased from approximately USD 4–5 per ounce in 1975 to over USD 40 per ounce by 2025. However, TELM results indicate that publication–patent–economic linkage strength is not directly proportional to silver price fluctuations.

From 1975 to the late 1980s, the number of publications remained relatively low, generally below 100 per year. During this period, silver prices experienced significant fluctuations, including a sharp peak around 1980 followed by a decline in the mid-1980s. TELM linkage intensities (*Lₜ_p_,ₖ)* remained weak during this phase, reflecting limited patent activity and low IPC/CPC diversification in silver halides technologies. Despite this volatility, research output remained limited, reflecting the dominance of traditional photographic and chemical applications of silver halides.

Between 1990 and 2005, publication output gradually increased to approximately 150–300 papers annually, indicating growing scientific interest. During this time, silver prices remained relatively stable with minor fluctuations. TELM results show a moderate increase in publication–patent coupling strength, particularly within emerging IPC/CPC categories linked to materials chemistry and optical coatings. This suggests that the increase in research activity was driven primarily by scientific and technological developments rather than economic factors.

A more pronounced growth phase emerged after 2005, when annual publications exceeded 400 by 2010. This trend coincided with rising silver prices and the rapid development of nanotechnology, advanced materials science, and photocatalysis, which expanded the application potential of silver halides beyond conventional imaging technologies. TELM analysis identifies a strong intensification of linkage strength within IPC/CPC classes related to nanostructured materials, photocatalysis, and Y02/Y04S low-carbon technologies, indicating technology-driven expansion.

From 2015 onward, research activity accelerated further, reaching approximately 700–1000 publications annually by the early 2020s. During the same period, silver prices showed moderate increases with several fluctuations, particularly after 2020. TELM results indicate that publication growth is strongly associated with increased patent activity in nanotechnology, environmental remediation, antimicrobial materials, and optical device technologies, rather than macroeconomic silver price trends.

The comparison indicates that although fluctuations in silver prices may influence research interest to some degree, TELM-based linkage analysis demonstrates that the primary driver of increased scientific output is the expansion of technological applications captured through IPC/CPC patent clustering and publication–patent network connectivity, particularly in nanotechnology, environmental engineering, and advanced optical materials.

### 3.2. Geographic and Organizational Trends in Silver Halides Research Publications

**Figure 5:**
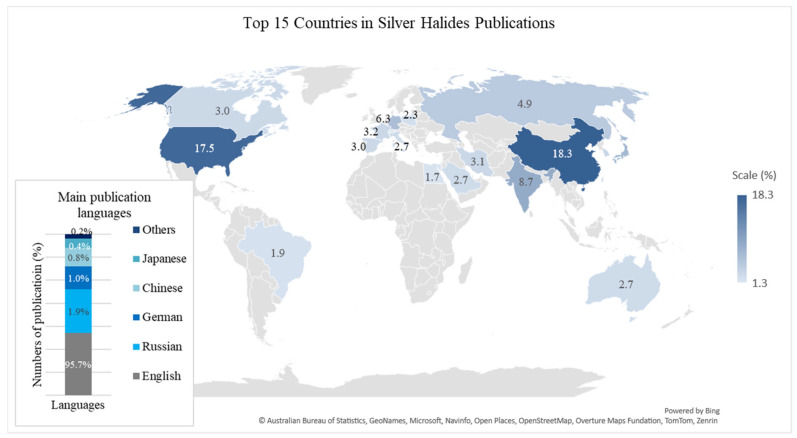
Global distribution of silver halides publications by the top 15 countries/regions and main publication languages based on WoS Core Collection (1975–2025).

The distribution of publications on silver halides across countries/regions, languages and affiliations, shown in Figure 5 and Table 1 reflect differences in scientific infrastructure, industrial demand, and historical strengths in materials chemistry and photochemistry. China (18.3%) and the United States (17.5%) lead in publication output due to their large research ecosystems, substantial government funding, and strong focus on nanomaterials, photocatalysis, and semiconductor research, where silver halides play a significant role. India (8.7%) also demonstrates a high contribution, which can be attributed to its rapidly expanding research sector and active studies in photocatalysis and environmental remediation. Several European countries show moderate but consistent contributions, including Germany (6.3%), Russia (4.9%), France (3.2%), Spain (3.0%), Italy (2.7%), and the United Kingdom (2.3%), reflecting their long-standing traditions in physical chemistry, crystallography, and materials science. Contributions from Iran (3.1%) and Saudi Arabia (2.7%) indicate increasing investment in scientific research and international collaboration, particularly in nanotechnology and environmental chemistry. Smaller shares are observed for Canada (3.0%), Australia (2.7%), and Brazil (1.9%), representing active but comparatively smaller research communities in this specialized field.

The inset further illustrates the main languages used in these publications, showing that English dominates with 95.7%, followed by Russian (1.9%), German (1.0%), Chinese (0.8%), Japanese (0.4%), and other languages (0.2%). The dominance of English facilitates global dissemination and collaboration in silver halides research. This distribution suggests that leadership and innovation in the field are concentrated in countries with strong funding systems and advanced research infrastructure, which may influence future technological developments, international collaborations, and knowledge dissemination in areas such as photocatalysis, environmental remediation, and advanced materials science.

**Figure 6:**
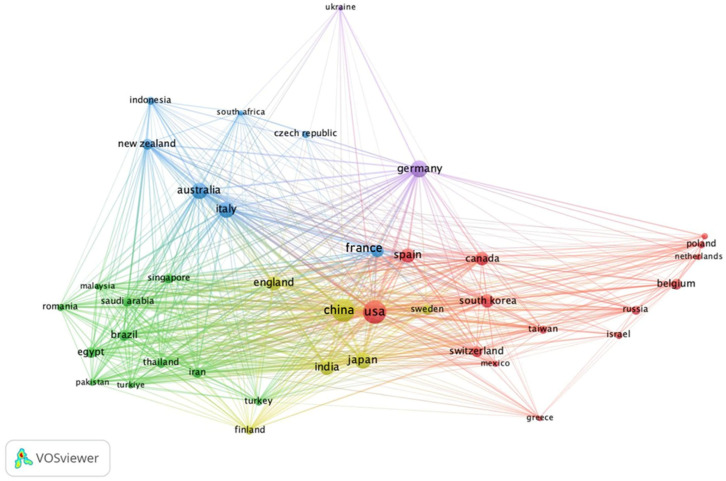
Cooperation network of leading countries in silver halides research based on WoS Core Collection (1975–2025), where node size reflects citation frequency and links indicate collaboration strength, showing five clusters: USA (red), China (yellow), Oceania–European (blue), developing countries (green), and a Germany-centered bridging group (purple), highlighting both dominant countries and global–regional collaborations.

Additionally, the cooperation network of leading countries in silver halides research (Figure 6) demonstrates a highly interconnected global collaboration system, in which node size reflects citation frequency and links represent the strength of cooperation between countries. The network is dominated by major research hubs such as the USA, China, and Germany, which occupy central positions and exhibit the highest citation impact and collaboration intensity.

Five distinct clusters can be identified. The yellow cluster, led by China and including India, Japan, and Nordic countries, highlights rapidly growing Asian research activity with expanding global connections. The red cluster, centered on the USA, integrates North America, Europe, and East Asia, representing the core of high-impact, transcontinental research. The blue cluster, comprising Australia, Italy, New Zealand, and related countries, indicates well-established collaborations between Europe and the Southern Hemisphere. The green cluster consists of developing and emerging research countries such as Malaysia, Saudi Arabia, Brazil, Egypt, Pakistan, Iran, and Türkiye, where collaboration is more regionally focused and citation impact is comparatively lower. Finally, the purple cluster, centered on Germany, acts as a key bridging node linking multiple regions and facilitating knowledge exchange across the global network.

Overall, the structure of the network reflects both strong regional clustering and extensive international integration, with leading countries playing a pivotal role in driving collaboration and scientific influence in silver halides research.

### 3.3. Leading Affiliations and Departments in Silver Halides Research

Table 1 demonstrates the analysis of global research output by affiliations. The Chinese Academy of Sciences (777 citations), Russian Academy of Sciences (647), and Centre National de la Recherche Scientifique (CNRS) (470) emerge as the leading institutions in terms of total citations. Other notable contributors include the Egyptian Knowledge Bank (EKB) (374), Indian Institute of Technology (IIT) System (302), and the University of California System (248). Corporate and governmental research organizations, such as Eastman Kodak (237), the United States Department of Energy (DOE) (230), and the Council of Scientific and Industrial Research (CSIR) India (207), further highlight the diversity of high-impact research across academic, industrial, and public sectors. Collectively, these affiliations reflect the global distribution of scientific influence and underscore the importance of large-scale, multidisciplinary research networks.

At the departmental level, substantial contributions are observed from the University of Hong Kong Faculty of Dentistry (172 citations), which leads research in dental and oral health. In the broader sciences, the King Saud University College of Science (110) and the Tel Aviv University Raymond and Beverly Sackler Faculty of Exact Sciences (107) demonstrate high-impact outputs in physics, chemistry, and mathematics. Specialized research units, including the King Abdulaziz University Faculty of Sciences (92) and the Tel Aviv University School of Physics and Astronomy (83), also make considerable contributions to global scientific literature. Additional leading laboratories include the Lomonosov Moscow State University Department of Chemistry (78), the University of Oxford Mathematical, Physical, and Life Sciences Division (78), and the University of Münster Department of Chemistry and Pharmacy (63). These departments and laboratories collectively emphasize the critical role of specialized research units in advancing high-impact science across multiple disciplines, including chemistry, physics, materials science, dentistry, and engineering.

All institutional metrics were unified to represent total citation counts derived from WoS-indexed publications associated with each affiliation.

**Figure 7:**
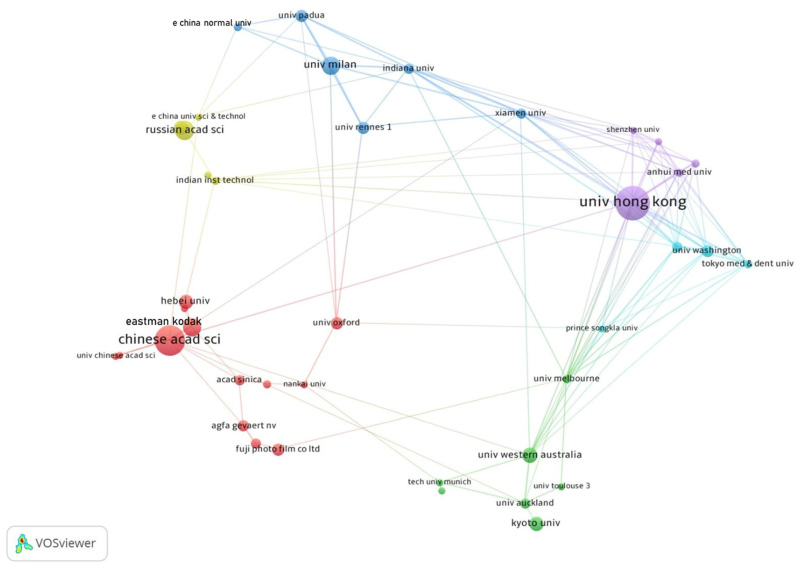
Collaborative network of leading institutions in silver halides research based on WoS Core Collection (1975–2025), where node size reflects citation impact, showing five distinct clusters: China–industry–UK axis (red), Russia-China–India collaboration group (yellow), European–US academic network (blue), Hong Kong–China hub (purple), Oceania–Asia–Europe network (green), and cyan (North America–Japan collaboration).

Figure 7 presents a collaborative network of leading universities and research institutions in silver halides research, illustrating collaboration strength, with the network organized into five color-coded clusters representing distinct collaborative communities:

The red cluster (China–industry–UK axis) centers on the Chinese Academy of Sciences as a highly influential node, strongly connected with Hebei University, UCAS, industrial partners Eastman Kodak, Agfa-Gevaert, and Fujifilm, as well as the University of Oxford and Nankai University, highlighting strong integration of academic and industrial research.

The yellow cluster (China–India collaboration group), anchored by the Russian Academy of Sciences and involving the University of Science and Technology of China and the Indian Institute of Technology, represents a Eurasian collaboration network emphasizing scientific cooperation between China, Russia, and India.

The blue cluster (European–US academic network) includes the University of Milan, University of Padua, Indiana University, University of Rennes 1, and East China Normal University, with the University of Milan showing high citation influence, reflecting strong transatlantic and European collaborations focused on fundamental research and material science.

The cyan cluster (North America–Japan collaboration) includes the University of Washington and Tokyo Medical and Dental University, highlighting collaborative ties between North American and Japanese institutions.

The purple cluster (Hong Kong–China hub), dominated by the University of Hong Kong, connects closely with Shenzhen University, Anhui Medical University, and Xiamen University, indicating a highly collaborative and regionally integrated network centered in Hong Kong and mainland China.

The green cluster (Oceania–Asia–Europe network), centered around the University of Western Australia and including the University of Melbourne, University of Auckland, Kyoto University, and Technical University of Munich, represents geographically diverse collaboration with moderate citation impact, collectively demonstrating a globally interconnected research landscape in silver halides with dominant hubs in China (Chinese Academy of Sciences), Hong Kong (University of Hong Kong), and Europe (University of Milan), notable industrial participation in the red cluster, and bridging institutions such as the University of Oxford and Xiamen University facilitating cross-cluster interactions and global knowledge exchange.

### 3.4. Analysis of WoS Subject Categories and Key Discipline-Pair Interactions

**Figure 8:**
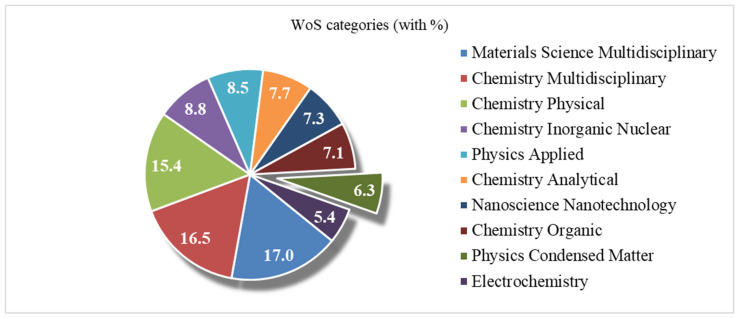
Distribution of the top 10 WoS categories for publications on silver halides (time span 1975–2025; unit, %).

Figure 8 presents the distribution of the top 10 Web of Science (WoS) categories for publications on silver halides. The dominant categories are Materials Science Multidisciplinary (17.0%), Chemistry Multidisciplinary (16.5%), and Physical Chemistry (15.4%), illustrating the interdisciplinary nature of silver halides research at the interface of materials science and chemistry. Significant contributions from Inorganic & Nuclear Chemistry (8.8%), Applied Physics (8.5%), and Analytical Chemistry (7.7%) reflect focused investigations into chemical composition, structural characterization, and application-driven physical properties. Research within Nanoscience and Nanotechnology (7.3%) and Organic Chemistry (7.1%) highlights expanding interest in nanoscale phenomena and molecular-level modifications. The highlighted segment for Applied Physics underscores its critical role in the development of silver halides–based technologies. Collectively, these findings confirm that silver halides research spans a broad spectrum of scientific disciplines, integrating fundamental chemical sciences, physics, and advanced materials engineering to support both theoretical understanding and technological innovation.

CDPI trends in Table 2 illustrate the temporal evolution of disciplinary contributions and key discipline-pair co-occurrences in silver halides research from 1975 to 2025. Table 2 presents descriptive indicators of interdisciplinary patterns, including normalized CDPI values and principal discipline-pair co-occurrence scores across the three study periods. The CDPI values show a steady increase across the three periods for most disciplines, indicating increasing interdisciplinary integration within the silver halides research field. For instance, Materials Science Multidisciplinary rose from 0.45 (1975–1999) to 0.60 (2000–2014) to 0.72 (2015–2025), while Chemistry Multidisciplinary increased from 0.48 to 0.63 to 0.70, and Physical Chemistry from 0.42 to 0.58 to 0.67. Inorganic & Nuclear Chemistry and Applied Physics followed similar trends, rising from 0.40 to 0.55 to 0.63 and 0.35 to 0.50 to 0.62, respectively. Emerging disciplines such as Nanoscience & Nanotechnology showed a more pronounced increase from 0.20 to 0.45 to 0.59, while Organic Chemistry grew from 0.18 to 0.40 to 0.52.

The key discipline-pair co-occurrence scores highlight strong interdisciplinary interactions. These scores represent normalized co-occurrence strengths derived from the frequency with which pairs of WoS subject categories appeared together within publication reference sets. Materials Science Multidisciplinary co-occurs most strongly with Chemistry Multidisciplinary (0.75), Nanoscience & Nanotechnology (0.70), Physical Chemistry (0.68), and Applied Physics (0.65). Chemistry Multidisciplinary is closely linked with Materials Science Multidisciplinary (0.75), Physical Chemistry (0.72), Inorganic & Nuclear Chemistry (0.69), and Analytical Chemistry (0.66). Physical Chemistry shows strong pairings with Chemistry Multidisciplinary (0.72), Inorganic & Nuclear Chemistry (0.70), and Applied Physics (0.68).

Collectively, the CDPI trends and discipline-pair linkages indicate that silver halides research has evolved into an increasingly interconnected field, characterized by strong interactions among chemistry, materials science, physics, analytical chemistry, and nanotechnology. These descriptive patterns demonstrate the broadening disciplinary scope of silver halides research over the past five decades.

### 3.5. Mapping Scientific Journals and Research Domains in Silver Halides Studies

**Figure 9:**
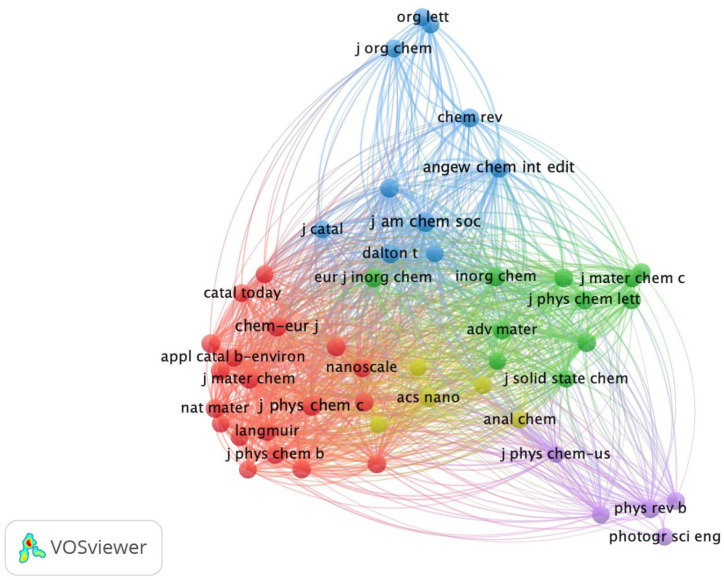
The network visualization of journals in silver halides research (WoS Core Collection, 1975–2025) shows citation-based relationships among journals, with node size reflecting citation impact, link strength indicating co-citation intensity, and five thematic clusters—catalysis and materials chemistry (red), organic and general chemistry (blue), inorganic and solid-state chemistry (green), nanoscience and nanotechnology (yellow), and physical chemistry and imaging science (purple)—with node size representing citation impact and connecting lines illustrating the interdisciplinary and integrative nature of the field.

This visualization generated by VOSviewer maps the network of academic journals based on citation relationships within silver halides research, shown in Figure 9. The journals cluster into five distinct groups, each color-coded to highlight thematic focus areas. The red cluster includes journals such as Catalysis Today, Applied Catalysis B: Environmental, Journal of Materials Chemistry, Nature Materials, and Langmuir, which emphasize catalysis, materials chemistry, and environmental applications. The blue cluster features Organic Letters, Journal of Organic Chemistry, Chemical Reviews, Angewandte Chemie International Edition, and Journal of the American Chemical Society, representing core organic and general chemistry research relevant to silver halides’ synthesis and molecular properties. The green cluster comprises Inorganic Chemistry, Journal of Materials Chemistry C, Journal of Physical Chemistry Letters, Advanced Materials, and Journal of Solid-State Chemistry, focusing on inorganic and solid-state chemistry, materials characterization, and analytical techniques. A smaller yellow cluster with journals like Nanoscale and ACS Nano highlights emerging research in nanoscience and nanotechnology, reflecting interest in nanoscale silver halides materials. The purple cluster, containing Physical Review B and Photographic Science and Engineering, represents physical chemistry and traditional imaging sciences, underscoring foundational physics and photographic applications of silver halides. Dense inter-cluster connections illustrate the interdisciplinary nature of silver halides research, with significant cross-citation among materials science, chemistry, and nanotechnology journals. Notably, high-impact journals such as Journal of the American Chemical Society, Chemical Reviews, Angewandte Chemie, and Nature Materials occupy central positions, reflecting their importance in disseminating key findings. Overall, the map reveals silver halides research as a multidisciplinary field integrating fundamental chemistry, materials science, nanotechnology, and imaging, demonstrating broad thematic diversity and strong interconnectedness across scientific domains.

### 3.6. Publishing Organizations and Funding Patterns in Silver Halides Research

The global patterns of silver halides–related publications from 1975 to 2025 reveal a high concentration among a limited number of publishers and funding agencies. As shown in Table 3, the top ten publishing organizations in the dataset are Elsevier (6735 publications), American Chemical Society (ACS, 2663), Wiley (2200), Springer Nature (1910), Royal Society of Chemistry (RSC, 1511), Taylor & Francis (780), MDPI (728), Mezhdunarodnaya Kniga (382), IOP Publishing Ltd. (341), and IS&T—Society for Imaging Science and Technology (336). Among these, Elsevier accounts for the largest share of publications, while IS&T and IOP Publishing primarily serve specialized research areas.

Regarding funding, the leading agencies are the National Natural Science Foundation of China (NSFC, 2331 publications), National Science Foundation (NSF, 535), U.S. Department of Health & Human Services (399), National Institutes of Health (NIH, USA, 382), Japan’s Ministry of Education, Culture, Sports, Science and Technology (MEXT, 364), Japan Society for the Promotion of Science (JSPS, 345), European Union (EU, 323), Grants-in-Aid for Scientific Research (KAKENHI, Japan, 316), Department of Science and Technology (DST, India, 314), and Fundamental Research Funds for the Central Universities (China, 279).

These data indicate that Elsevier is the largest global publisher, and NSFC represents the most prolific funding source. Other publishers and agencies make important contributions within specialized disciplines or regional contexts, highlighting both the concentrated and diverse patterns of publication and funding in silver halides research over the past five decades.

## 4. Discussion

### 4.1. Natural and Synthetic Silver Halides: Sources and Processing

Silver halides occur from two main sources: natural formation as secondary minerals in the oxidized zones of silver-rich deposits and artificial production through laboratory or industrial synthesis (see Table 4). Natural silver halides minerals are not reported separately in global mineral statistics because they typically occur as supergene phases within polymetallic and precious-metal systems, reflecting near-surface enrichment of silver during weathering, particularly in arid environments [26,27]. Global silver reserves are estimated at ~640,000 metric tons, with major contributions from Peru, Australia, Russia, China, Poland, Mexico, Chile, the United States, and Kazakhstan [28]. Within these deposits, silver halides—mainly chlorargyrite (AgCl), bromargyrite (AgBr), iodargyrite (AgI), and mixed halides such as embolite—form during oxidation of primary silver sulfides (e.g., acanthite/argentite) through interaction with halogen-bearing fluids.

Chlorargyrite is the most common silver halides and occurs in oxidized zones of major silver districts including Broken Hill, Arkharly, the western United States, Mexico, Chile, Germany, and Bolivia, commonly associated with native silver and other supergene minerals. Bromargyrite is typical of arid to semi-arid environments such as Mexico, Chile, Arizona, and Broken Hill, where bromine-rich fluids promote its formation. Iodargyrite is rarer but has been reported in Australia, Mexico, Chile, Kazakhstan, and Europe in iodine-bearing systems (iodargyrite, n.d.). Their stability is strongly climate-dependent, with arid conditions favoring preservation, while humid environments promote dissolution or transformation [27]. In some gossans (e.g., Almanda, Australia), silver halides may even dominate near-surface silver mineralization [29,30,31,32,33,34,35]. This spatial variability in silver halide formation and preservation provides important context for interpreting their geochemical evolution and is consistent with bibliometric keyword clusters emphasizing ‘supergene processes’, ‘oxidation zones’, and ‘ore weathering’ trends.

In addition to natural formation, silver halides are widely synthesized by precipitation reactions between silver salts (e.g., AgNO_3_) and halides salts (NaCl, KBr, KI), producing AgCl, AgBr, and AgI. Controlled synthesis (e.g., temperature, stirring, stabilizers like gelatin or polymers) enables precise control of crystal size and morphology, widely used in photographic and optical materials. These synthetic methods also help simulate natural supergene processes of silver halides formation in oxidized ore systems [26,27,36,37,38,39].

**Figure 10:**
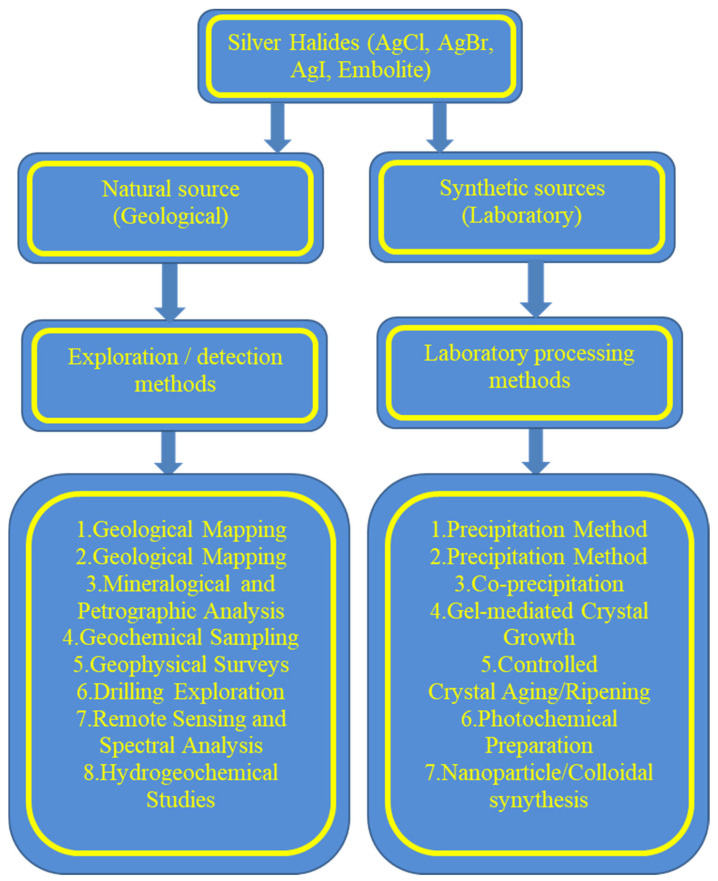
Overview of the sources and processing of silver halides.

To integrate mineralogical and synthetic perspectives with bibliometric outcomes, a conceptual framework is introduced (Figure 10). This framework connects natural occurrence of silver halides in supergene environments, laboratory synthesis routes, crystal structure and morphology, physicochemical properties, and application domains such as imaging, optoelectronics, and photocatalysis. Furthermore, CDPI and TELM analyses, suggest a shift in publication themes from geology-centered research (e.g., ore deposits and oxidation processes) toward materials science and nanotechnology-related topics within the bibliometric dataset, highlighting the interdisciplinary evolution of silver halides research.

Several methods are employed in the detection and production of silver halides, both naturally occurring and synthetically produced (see Figure 10). Geological exploration and detection involve detailed field geological mapping of oxidation zones and gossans, mineralogical and petrographic analysis through optical microscopy, scanning electron microscopy (SEM), and X-ray diffraction (XRD) to confirm silver halides phases, as well as geochemical sampling of soils, sediments, and rocks to detect silver and halides enrichments. From a bibliometric perspective, TELM-based publication–technology linkages and CDPI-derived interdisciplinarity scores suggest increasing connections among publications related to analytical mineralogy, nanomaterial synthesis, and optoelectronic research topics. However, these relationships reflect patterns in the scientific literature and should not be interpreted as direct evidence of technological implementation or industrial impact.

Geophysical surveys including induced polarization (IP), resistivity, and magnetic methods identify sulfide mineralization and structural controls, while drilling exploration provides subsurface samples to delineate oxidation profiles. Remote sensing and hyperspectral spectral analysis further aid in detecting alteration minerals indicative of silver halides, and hydrogeochemical studies analyse halides ions in groundwater to understand fluid sources responsible for halides mineral formation [40,41,42].

In contrast, synthetic silver halides are prepared in the laboratory primarily by precipitation methods, where soluble silver salts like silver nitrate (AgNO_3_) react with halides salts (NaCl, KBr, KI) to form insoluble AgCl, AgBr, or AgI precipitates, with co-precipitation techniques used to produce mixed halides compounds such as embolite. Controlled crystal growth in gel media allows uniform nucleation and microcrystal formation, while aging or ripening processes refine crystal size and quality. Photochemical preparation exploits the photosensitivity of silver halides for applications in photography and optics, and advanced nanoparticle or colloidal syntheses employ stabilizing polymers or surfactants to produce silver halides nanoparticles for sensors and optical devices. These combined geological and synthetic methods provide comprehensive approaches to understanding, detecting, and producing silver halides for both natural resource exploration and technological applications [27,38,43,44,45,46].

### 4.2. Silver Halides in Natural and Synthetic Forms: Functional Comparisons and Applied Implications

Silver halides—encompassing mineral forms such as silver chloride (AgCl), silver bromide (AgBr), silver iodide (AgI), and synthetic analogs—represent a class of light-sensitive inorganic compounds with extensive technological and mineralogical relevance. CDPI results further suggest a growing interdisciplinarity between geosciences and materials engineering, reflecting increasing publication linkages between mineral occurrence studies and functional materials research within the literature. These phases exhibit distinctive physicochemical properties, including tunable crystallography, phase stability, and optical responsiveness, which underpin their multifunctional applications in sensors, optoelectronics, photocatalysis, and biomedical coatings. From a practical perspective, information on silver halides can be accessed through mineralogical databases, WoS platform, and materials science repositories, which together provide integrated geological, chemical, and application-oriented datasets.

In comparison, mineral occurrences are often limited and heterogeneous, which constrains direct industrial use, whereas synthetic forms are widely employed in technological applications that demand uniformity and reproducibility. Mineral forms provide a geological and mineralogical context, useful for studying trace element incorporation, crystallography, and formation processes. Synthetic forms enable technological exploitation, allowing integration into advanced devices where specific size, shape, and surface characteristics are critical. The importance of silver halides extends across multiple high-impact application domains, including medical imaging (e.g., X-ray and radiographic films), astrochemistry and astrophotography (light-sensitive detection of faint celestial signals), and advanced light detection systems such as photonic sensors, optical imaging devices, and radiation detectors.

Table 5 compares mineral and synthetic silver halides across key aspects. Mineral silver halides are naturally occurring, often limited in availability, and may contain impurities, which restricts their structural control and functionalization. In contrast, synthetic silver halides offer high purity, precise control over crystal size and morphology, and greater flexibility for technological applications, although they require higher production costs. Overall, minerals provide a geological context, while synthetic forms enable tailored, multifunctional materials for advanced devices.

Literature-based implications and research perspectives. The following implications are derived from the reviewed scientific literature and are presented as research perspectives. They should not be interpreted as conclusions directly supported by the bibliometric analyses: (1) Understanding mineral occurrences may help inform potential extraction strategies and sustainable resource-use approaches; (2) Synthetic silver halides provide opportunities for developing multifunctional materials for optoelectronic, plasmonic, and photocatalytic applications; (3) Previous mineralogical studies have reported that silver halides can host trace metals (e.g., Au, Ag, and Pb), which may influence material properties and could have implications for resource recovery; and (4) Synthetic production can provide high-purity and application-specific materials while reducing dependence on natural mineral sources, although production costs and energy requirements may be higher. (5) The broad technological relevance of silver halides is further evidenced by their long-standing use in imaging science, astronomical observation, and photon detection technologies, highlighting their continued importance across both fundamental and applied research domains.

### 4.3. Silver Halides as Potential Resource Materials: Occurrence, Extraction, and Recovery

This section provides literature-based context regarding the occurrence, extraction, and recovery of silver halides. The discussion complements the bibliometric findings but is not derived directly from the WoS bibliometric dataset.

Silver halides (AgCl, AgBr, AgI) have been discussed in the literature as a potentially important secondary source of silver under specific geological and technological condition, particularly in halogen-rich environments where silver-bearing systems interact with chloride-, bromide-, or iodide-bearing fluids. While traditionally studied for their optical and electronic properties, increasing attention is being given to their significance in resource sustainability and circular economy frameworks.

In natural systems, silver halides occur as rare minerals such as chlorargyrite, bromargyrite, and iodargyrite, mainly forming in oxidized zones of silver-rich ore deposits. Their formation is controlled by halogen availability, redox conditions, and fluid composition.

In supergene settings, silver released from primary sulfides reacts with halides-bearing meteoric or basinal fluids, precipitating stable silver halides under oxidizing and saline conditions, particularly in arid or evaporitic environments where chloride-rich brines enhance silver transport and re-precipitation [13].

In sedimentary and coal-bearing basins, silver halides may also form during diagenesis through interactions between silver, organic matter, and halogen-enriched pore waters. Organic matter can act as both a reductant and ligand source, promoting silver mobility and localized precipitation, especially in chlorine- or iodine-rich environments relevant to coal and organic-rich systems [13,14,15,16,17,18,19,20,21,22,23,24,25,26,27].

The extraction of silver from halides phases differs fundamentally from conventional sulfide or native silver processing. Silver halides are generally less amenable to direct cyanidation due to their low solubility and kinetic limitations [27,40]. Instead, alternative approaches are required, including:

1. Photochemical and thermal decomposition, where exposure to light or heat induces breakdown of Ag–X bonds, producing metallic silver.

2. Hydrometallurgical methods, such as ammoniacal leaching or thiosulfate systems, which enhance silver solubility through complexation.

3. Chloride-based leaching systems, which can both dissolve and re-precipitate silver depending on solution chemistry, requiring careful control of Eh–pH conditions.

These methods are particularly relevant in processing residues from photographic waste, electronic materials, and industrial catalysts, where silver halides are abundant in synthetic form.

The literature identifies secondary resource recovery as a potential area of interest for silver halides. Historically, photographic films and papers represented a significant reservoir of AgBr and AgCl, and established recovery processes (e.g., chemical reduction, electrolysis) have demonstrated high efficiency [13,41]. Although digital technologies have reduced this source, other emerging reservoirs include:

1. Electronic and optoelectronic waste, where silver halides are used in sensors and coatings.

2. Industrial catalysts and antimicrobial materials, particularly AgCl-based compounds.

3. Nanomaterials and functional composites, where engineered silver halides are incorporated for photocatalytic or antibacterial properties.

Recovery from these materials aligns with circular economy principles, reducing dependence on primary mining and mitigating environmental impacts associated with silver extraction. From an environmental perspective, silver halides exhibit relatively low solubility under neutral conditions but may release silver ions under changing redox or photochemical conditions. This dual behavior necessitates careful management during extraction and disposal. Economically, the viability of silver halides as a resource depends on their concentration, accessibility, and co-occurrence with other valuable elements (e.g., Au, Pb, Zn). In low-grade geological settings, such as coal or sedimentary basins, their significance may lie more in trace element recovery and by-product extraction rather than as primary targets [42,43,44,45,46].

### 4.4. Silver Halides in Science and Technology: Innovations and Progress over Five Decades (1975–2025)

Over the five decades from 1975 to 2025, silver halides have undergone continuous innovation, technological refinement, and sector-specific evaluation, evolving from traditional photographic materials to advanced applications in medicine, environmental science, and nanophotonics. During this period, silver halides demonstrated significant adaptability, maintaining relevance across multiple sectors beyond conventional photographic applications (Table 6).

In the photographic domain, the period 1975–1985 was marked by the introduction of tabular grain (T-grain) emulsions, representing a major advancement in crystal engineering. These high-aspect-ratio grains enhanced light absorption efficiency, resulting in higher sensitivity, finer grain, and sharper images, which established the foundation for professional and consumer photography [47,48].

Between 1985 and 2000, research on silver halides focused on dye coupler chemistry and high-resolution emulsions, complemented by holographic recording techniques using Silver Halides Sensitized Gelatin (SHSG). Dye couplers significantly improved color stability and archival permanence in color negative and print films, while high-resolution and ultra-fine grain emulsions enabled holographic and optical applications with exceptional detail and minimal noise [49,50,51].

The early 21st century (2000–2015) saw the hybridization of silver halides with digital imaging technologies, where traditional emulsions were adapted as printing media for digitally captured images. This era emphasized improved processing workflows that preserved tonal qualities and archival characteristics, bridging the gap between analog chemistry and digital imaging [52]. From 2015 to 2025, research shifted toward sustainable production methods and nanostructured silver halides, incorporating green gelatin alternatives, environmentally friendly chemical practices, and advanced nanoscale optical applications [53].

Beyond photography, silver halides have played a pivotal role in medicine and biomedical applications. AgBr and AgCl emulsions, as well as nanoparticle formulations, have been used in high-resolution X-ray films, antimicrobial coatings for wound dressings and implants, and experimental photoactivated drug delivery systems for photodynamic therapy [54,55].

In environmental and climate applications, silver iodide (AgI) has been extensively applied in cloud seeding. Advances in nanostructured AgI crystals have enhanced ice nucleation efficiency while reducing ecological impacts, thereby improving the safety and effectiveness of precipitation enhancement operations [56,57].

Silver halides have also contributed significantly to photocatalysis and environmental remediation. AgCl, AgBr, and Ag/AgX composites have been employed to degrade organic pollutants and industrial dyes in water treatment. Integration with TiO_2_ or graphene in hybrid nanocatalysts has further enhanced photocatalytic efficiency by improving light absorption and electron-hole separation [58,59,60].

In electronics and optoelectronics, nanosilver halides have been utilized for holographic data storage and plasmonic sensors, exploiting tunable optical properties and nanoscale precision for advanced photonic applications [61,62].

Looking toward 2025 and emerging technologies, recent publications have reported research exploring ultra-fine nanostructured silver halides and hybrid quantum-photonic emulsions are being explored for quantum sensing, ultrahigh-resolution imaging, and eco-friendly photonics. These developments indicate that silver halides will continue to play a significant role in cutting-edge scientific and technological domains [63,64,65,66,67,68,69]. These emerging directions are based on trends reported in the scientific literature and should not be interpreted as predictions of future technological adoption or market development.

### 4.5. Core Disciplines Driving the Growth of Silver Halides Research

As indicated by the CDPI, silver halides research has increasingly evolved into a highly interdisciplinary field between 1975 and 2025. Materials Science Multidisciplinary serves as the central hub (CDPI = 0.72), closely connected with Chemistry Multidisciplinary (0.70), Physical Chemistry (0.67), and Applied Physics (0.62), reflecting the integration of materials characterization, chemical synthesis, photophysical studies, and device-oriented research. Chemistry and Physical Chemistry remain key drivers of fundamental understanding, supporting innovations in crystal engineering, photochemical processes, and structural analysis, while Applied Physics and Nanoscience & Nanotechnology underpin applied developments in optical devices, nanoscale materials, and imaging technologies. Supporting disciplines, including Electrochemistry, Inorganic Chemistry, and Organic Chemistry, provide essential mechanistic and synthetic foundations that enable hybrid material design, functional modifications, and advanced applications. Together, these disciplines demonstrate that silver halides research spans fundamental science, applied chemistry, and materials engineering, illustrating a convergence of theoretical and practical innovation.

The journal co-citation network underscores the interdisciplinary nature of silver halides research, as demonstrated in the Results section. Key journals include Catalysis Today, Applied Catalysis B: Environmental, Journal of Materials Chemistry, Nature Materials, and Langmuir, reflecting catalysis, material synthesis, and environmental applications; Organic Letters, Journal of Organic Chemistry, Chemical Reviews, Angewandte Chemie International Edition, and Journal of the American Chemical Society, representing organic and general chemistry; Inorganic Chemistry, Journal of Physical Chemistry Letters, Advanced Materials, and Journal of Solid-State Chemistry, highlighting inorganic, solid-state chemistry, and materials characterization; Nanoscale and ACS Nano, illustrating nanoscience and nanoscale applications; and Physical Review B and Photographic Science and Engineering, emphasizing physical chemistry and imaging science.

These findings illustrate that silver halides research has matured into a multidimensional field where materials science, chemistry, physics, and nanotechnology converge. The increasing prominence of journals in catalysis, nanoscience, and applied materials suggests increased publication activity related to functional applications, while fundamental studies in photochemistry and solid-state properties continue to provide critical mechanistic insights. This pattern underscores how silver halides research addresses fundamental scientific questions while being increasingly associated with publications related to optics, imaging, photocatalysis, and environmental applications. Bibliometric evidence alone cannot establish the extent of technological, economic, or societal impact (Table 7).

**Figure 11:**
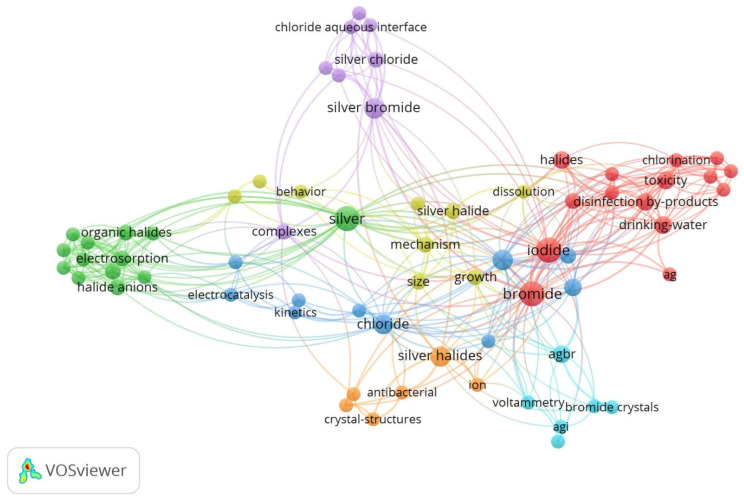
Keyword co-occurrence network of silver halides–related publications, (WoS Core Collection, 1975–2025), where node size represents keyword frequency and colors highlight major thematic clusters: red (environmental and water chemistry), green (electrochemical and catalytic processes), yellow (crystal growth mechanisms), orange (structural and antibacterial properties), blue/cyan (electroanalytical studies), and purple (interfacial chemistry).

The keyword co-occurrence network (Figure 11), visualized using VOSviewer, provides clear evidence of the thematic structure and interdisciplinary nature of research related to silver halides and halides chemistry. The network illustrates how fundamental chemistry, environmental science, materials science, and electrochemical studies intersect within this research field. The visualization reveals six major thematic clusters, indicating the main research directions in this field.

The red cluster represents the environmental and water chemistry aspects of halides research, characterized by keywords such as bromide, iodide, halides, chlorination, toxicity, disinfection by-products, drinking water, and Ag. This cluster reflects studies investigating the environmental behavior of halides ions during water treatment processes, particularly the transformation of bromide and iodide during chlorination and the formation of potentially harmful disinfection by-products in drinking water systems.

The green cluster is associated with electrochemical and catalytic processes involving halides and silver, including keywords such as organic halides, electrosorption, electrocatalysis, halides anions, complexes, and silver. This cluster represents research focused on electrochemical reactions, adsorption mechanisms, and catalytic transformations of halides-containing compounds, highlighting the role of silver in electrochemical and catalytic systems.

The yellow cluster highlights fundamental physicochemical processes related to silver halides formation and transformation, including keywords such as silver halides, mechanism, growth, size, dissolution, and behavior. These studies primarily address nucleation and crystal growth mechanisms, particle size effects, and dissolution processes that control the formation and stability of silver halides materials.

The orange cluster focuses on structural and functional properties of silver halides, represented by keywords such as crystal structures, antibacterial, ion, and silver halides. This cluster reflects investigations of crystallographic characteristics and functional properties of silver halides materials, including their antimicrobial behavior and ionic interactions.

The blue and cyan clusters emphasize electroanalytical and materials characterization studies, including keywords such as AgBr, AgI, bromide crystals, voltammetry, chloride, kinetics, and ion. These clusters represent research employing electrochemical and analytical techniques to investigate the physicochemical properties and electrochemical behavior of silver halides crystals and halides ions.

Finally, the purple cluster represents interfacial and aqueous chemistry of silver halides, with keywords such as silver chloride, silver bromide, and chloride aqueous interface. This cluster highlights studies examining precipitation, dissolution, and solid–liquid interfacial processes governing the formation and transformation of silver halides compounds in aqueous environments.

The strong interconnections among clusters demonstrate the interdisciplinary nature of silver halides research, integrating environmental chemistry, electrochemistry, materials science, and analytical chemistry. The central position of keywords such as silver, bromide, iodide, and chloride further emphasize their fundamental role in controlling the chemical behavior, environmental implications, and technological applications of silver halides systems.

### 4.6. Sustainability and Future Direction

**Figure 12:**
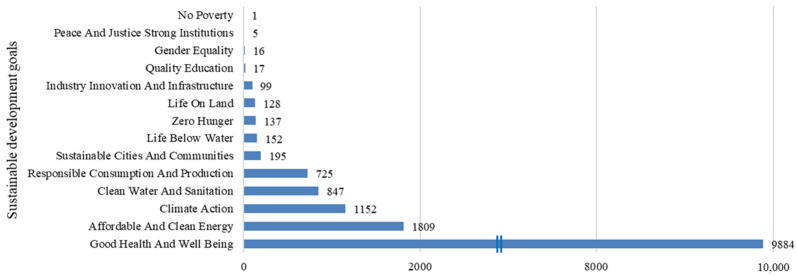
Distribution of research publications across Sustainable Development Goals based on WoS data, highlighting disparities in focus areas from 1975 to 2025.

Figure 12 illustrates the distribution of research publications and projects related to the United Nations Sustainable Development Goals (UN SDGs) from 1975 to 2025, based on Web of Science data. The analysis reveals notable disparities in research focus across different SDGs. According to WoS SDG classifications, Good Health and Well-Being emerges as the most extensively studied goal, with 9884 publications, highlighting the global prioritization of health-related challenges and biomedical applications, including sensor technologies. This is followed by Affordable and Clean Energy (1809 publications) and Climate Action (1152 publications), reflecting significant scientific attention toward energy-efficient, sustainable, and environmentally responsive sensor systems.

Moderate research activity is observed in Clean Water and Sanitation (847 publications) and Responsible Consumption and Production (725 publications), indicating active engagement with water quality monitoring, environmental sensing, and sustainable industrial applications. A secondary tier of SDGs, including Sustainable Cities and Communities (195 publications), Life Below Water (152), Zero Hunger (137), Life on Land (128), and Industry, Innovation and Infrastructure (99), demonstrates comparatively lower, yet meaningful, research contributions in urban environmental monitoring, ecosystem and agricultural sensors, and industrial sensing technologies.

Conversely, social and governance-oriented goals such as Quality Education (17 publications), Gender Equality (16), and Peace and Justice Strong Institutions (5) receive considerably less attention. No Poverty shows the lowest research activity with a single publication, suggesting a critical gap in scholarly focus on poverty-alleviation applications within this dataset.

These findings indicate that publications in the dataset are more frequently associated with health, energy, and environmental SDG categories than with other SDGs. These publication patterns identify areas of research emphasis within the literature; however, they should not be interpreted as direct evidence of societal impact or technological effectiveness.

Future research is expected to integrate materials design with sustainability considerations, particularly in energy and environmental technologies. Bibliometric patterns suggest increasing publication activity related to nanotechnology, optoelectronics, photocatalysis, sustainability, and sensing-oriented topics compared with traditional photographic applications. Early work focused on emulsion chemistry, grain size control, and light-sensitive imaging, whereas current research emphasizes nanotechnology, optoelectronics, photocatalysis, and advanced sensor platforms, reflecting broader trends in materials science and functional device development.

A major emerging area is the development of nanostructured silver halides, which exhibit tunable optical, electronic, and catalytic properties. These nanoscale materials are increasingly applied in photocatalytic pollutant degradation, plasmonic and optical sensing, high-resolution holography, and environmental monitoring. Hybrid and composite systems, including silver halides–perovskite and polymer-based materials, demonstrate the potential for flexible, multifunctional sensors and optoelectronic devices. Research in this area highlights the importance of interface engineering, nanoscale morphology control, and long-term material stability for practical applications.

Sustainable and environmentally friendly synthesis represents another key frontier. Methods such as biogenic production using plant or microbial extracts, solvent-free or water-based approaches, and recyclable silver recovery are gaining attention, aligning material fabrication with green chemistry and sustainable sensor development. Silver halides also show promise in energy and photonic applications, including solar-driven water splitting, CO_2_ reduction, and antimicrobial coatings, further connecting sensor development with environmental sustainability.

Bibliometric trends reveal increasing integration of computational modelling and artificial intelligence for predictive design of silver halides materials, facilitating rational design of nanostructured sensors and functional devices. By linking historical discoveries with these emerging technologies, future research is expected to balance fundamental chemistry, innovative sensor development, and environmental responsibility, positioning silver halides at the forefront of advanced materials science, multifunctional sensors, and applied photonic technologies.

Overall, the future directions discussed in this section are informed by trends observed in the scientific literature and bibliometric patterns. They should be considered research opportunities and emerging themes rather than direct evidence of industrial adoption, market potential, economic value, or societal impact.

## 5. Conclusions

This study presents the first systematic bibliometric analysis of global silver halides research over five decades (1975–2025), based on 23,841 Web of Science publications. By integrating the Cross-Disciplinary Publication Index (CDPI), Technology–Economic Linkage Model (TELM), VOSviewer, and Excel, we elucidated the evolution, interdisciplinary characteristics, and emerging frontiers of silver halides science.

Research on silver halides has undergone substantial transformation. Historically dominated by photographic applications, the field has diversified into optical and plasmonic sensors, biomedical diagnostics, photocatalytic systems, and sustainable technologies. The combined bibliometric evidence indicates a sustained shift toward functional materials engineering and nanotechnology-driven applications.

The findings demonstrate that silver halides have evolved into strategically important multifunctional materials with growing relevance to sensing, environmental remediation, and advanced photonic technologies. Interdisciplinary integration is strongest in Materials Science Multidisciplinary (CDPI 0.72), Chemistry Multidisciplinary (0.70), and Physical Chemistry (0.67), while Nanoscience and Nanotechnology exhibited the most pronounced growth (0.20 → 0.59), reflecting a strategic shift toward nanoscale phenomena and functional sensor applications. The coexistence of natural and synthetic silver halides further highlights their significance as both mineral resources and model materials for structure–property investigations.

Several limitations should be acknowledged. The analysis is based exclusively on publications indexed in the Web of Science Core Collection and may only partially reflect the broader scope of relevant research reported outside this database. In addition, bibliometric indicators identify research trends and relationships but do not independently validate scientific quality, technological performance, or application outcomes.

Future studies should therefore integrate multiple databases and complementary approaches, including systematic reviews, patent analyses, and experimental validation, to verify emerging research directions and technology-development pathways. Continued investigation of nanostructured and hybrid silver halide systems, particularly for photocatalysis, optical sensing, plasmonic devices, and energy-related applications, will help assess their practical potential and long-term technological impact.

## Figures and Tables

**Table 1:** Global research output by the top 20 affiliations and departments, highlighting total citations and leading research on silver halides.

No.	Affiliation	Cited	No.	Affiliation with Department	Cited
1	Chinese Academy of Sciences	777	1	The University of Hong Kong Faculty of Dentistry	172
2	Russian Academy of Sciences	647	2	King Saud University College of Science	110
3	Centre National De La Recherche Scientifique Cnrs	470	3	Tel Aviv University Raymond And Beverly Sackler Faculty of Exact Sciences	107
3	Egyptian Knowledge Bank Ekb	374	3	King Abdulaziz University Faculty of Sciences	92
3	Indian Institute of Technology System Iit System	302	3	Tel Aviv University School of Physics and Astronomy	83
6	University of California System	248	6	Lomonosov Moscow State University Department of Chemistry	78
7	Eastman Kodak	237	7	University of Oxford Mathematical Physical and Life Sciences Division	78
7	United States Department of Energy Doe	230	7	University of Münster Department of Chemistry and Pharmacy	63
9	Council of Scientific Industrial Research Csir India	207	9	University of Science and Technology of China School of Chemistry and Materials Science	61
10	University of Hong Kong	206	10	Jilin University College of Chemistry	57
11	Tel Aviv University	193	11	Xiamen University College of Chemistry and Chemical Engineering	56
12	University of Chinese Academy of Sciences Cas	192	12	Islamic Azad University Sahreza Branch	53
12	Cnrs Institute of Chemistry Inc	184	12	Islamic Azad University Sahreza Branch Department of Chemistry	52
14	King Saud University	177	14	Warsaw University Faculty of Chemistry	52
14	Consiglio Nazionale Delle Ricerche Cnr	161	14	King Saud University Department of Chemistry	51
16	Consejo Superior De Investigaciones Cientificas Csic	156	16	Nankai University College of Chemistry	50
16	University System of Ohio	146	16	University of Padua Department of Chemistry	49
16	Swiss Federal Institutes of Technology Domain	140	16	University of Padua School of Sciences	49
19	United States Department of Defense	136	19	University of Oxford Department of Chemistry	47
20	Islamic Azad University	135	20	Guru Nanak Dev University Faculty of Sciences	43

**Table 2:** Descriptive indicators of interdisciplinary patterns in silver halides research (1975–2025), showing normalized CDPI values and principal discipline-pair co-occurrence scores.

Main Discipline	CDPI Periods (1975–1999/2000–2014/2015–2025)	Top Discipline Pairs (CDPI Score)
Materials Science Multidisciplinary	0.45/0.60/0.72	Chemistry Multidisciplinary (0.75), Nanoscience Nanotechnology (0.70), Chemistry Physical (0.68), Applied Physics (0.65)
Chemistry Multidisciplinary	0.48/0.63/0.70	Materials Science Multidisciplinary (0.75), Physical Chemistry (0.72), Inorganic & Nuclear Chemistry (0.69), Analytical Chemistry (0.66)
Physical Chemistry	0.42/0.58/0.67	Chemistry Multidisciplinary (0.72), Inorganic & Nuclear Chemistry (0.70), Applied Physics (0.68)
Inorganic & Nuclear Chemistry	0.40/0.55/0.63	Physical Chemistry (0.70), Chemistry Multidisciplinary (0.69), Organic Chemistry (0.60)
Applied Physics	0.35/0.50/0.62	Materials Science Multidisciplinary (0.65), Physical Chemistry (0.68), Analytical Chemistry (0.64)
Analytical Chemistry	0.33/0.48/0.60	Chemistry Multidisciplinary (0.66), Applied Physics (0.64), Organic Chemistry (0.58)
Nanoscience Nanotechnology	0.20/0.45/0.59	Materials Science Multidisciplinary (0.70), Chemistry Organic (0.55)
Chemistry Organic	0.18/0.40/0.52	Inorganic & Nuclear Chemistry (0.60), Analytical Chemistry (0.58), Nanoscience Nanotechnology (0.55)
Electrochemistry	0.15/0.35/0.45	Applied Physics (0.55), Physical Chemistry (0.50)

**Table 3:** Top 10 Publishing Organizations and Leading Funding Agencies for Silver Halides–Related Research (1975–2025).

No.	Publishing Organization	Publications	Funding Agencies	Publications
1	Elsevier	6735	National Natural Science Foundation of China NSFC	2331
2	Amer Chemical Soc	2663	National Science Foundation NSF	535
3	Wiley	2200	United States Department of Health Human Services	399
4	Springer Nature	1910	National Institutes of Health NIH USA	382
5	Royal Soc Chemistry	1511	Ministry of Education Culture Sports Science and Technology Japan MEXT	364
6	Taylor & Francis	780	Japan Society for the Promotion of Science	345
7	Mdpi	728	European Union EU	323
8	Mezhdunarodnaya Kniga	382	Grants in Aid for Scientific Research KAKENHI	316
9	Iop Publishing Ltd.	341	Department of Science Technology India	314
10	I S & T—SOC Imaging science technology	336	Fundamental Research Funds for the Central Universities	279

**Table 4:** Natural occurrence and laboratory synthesis sources of silver halides.

Mineral/Source Type	Typical Geological or Laboratory Setting	Major Occurrence/Production Examples
Chlorargyrite (AgCl)	Oxidized zones of silver veins, supergene enrichment	Western USA (Nevada, Arizona), Broken Hill (Australia), Chile (Huantajaya), Mexico (Zacatecas, Plateros), Kazakhstan (Arkharly), Germany, Bolivia
Bromargyrite (AgBr)	Highly oxidized, arid conditions, supergene zones	Plateros (Mexico), Chile (Atacama), Bisbee (Arizona, USA), Broken Hill (Australia), Kazakhstan (Arkharly)
Iodargyrite (AgI)	Rare supergene, oxidized occurrences, arid climates	Australia (Broken Hill), Mexico (Plateros), Chile, Kazakhstan (Arkharly)
Embolite/Chlorobromides	Supergene assemblages in arid to semi-arid oxidized zones	Western USA (Nevada, Utah), Australia, Mexico, Kazakhstan (Arkharly), Chile
Synthetic Silver Chloride	Laboratory precipitation from AgNO_3_ + NaCl solutions	Produced in chemical laboratories and industrial photographic emulsions
Synthetic Silver Bromide	Controlled precipitation in gelatin or polymer media	Photographic film and imaging materials
Synthetic Silver Iodide	Laboratory synthesis via AgNO_3_ + KI reaction	Cloud seeding materials, optical and photosensitive compounds
Synthetic Mixed Silver Halides	Co-precipitation and crystal growth in controlled solutions	Photographic emulsions and optical sensors

**Table 5:** Comparative advantages and disadvantages.

Aspect	Mineral Silver Halides	Synthetic Silver Halides
Availability	Limited, dependent on mineral deposits	Scalable, reproducible in laboratories and industry
Purity	Often contains inclusions, trace elements	High purity, controlled composition
Structural Control	Limited control over morphology and size	Precise control of crystal size, shape, and surface properties
Functionalization	Difficult to modify without affecting structure	Easily modified for sensors, photocatalysts, and biomedical applications
Cost	Lower extraction cost if deposit is accessible	Higher production cost due to processing
Application Flexibility	Mainly photographic and imaging	Multifunctional: optical sensors, plasmonics, photocatalysis, coatings

**Table 6:** Evaluative trends in a five-decade review of silver halides science: Key discoveries, technological innovations.

Era/Sector	Silver Halides Type/Form/Innovation	Applications/Impact
1975–1985: Photography	Tabular grain (T-grain) emulsions, improved crystal engineering	Higher sensitivity, finer grain, sharper images; foundation for professional photography
1985–2000: Photography	Dye coupler chemistry, high-resolution and ultra-fine grain emulsions, holographic SHSG techniques	More stable color images, holography, optical recording, scientific imaging
2000–2015: Photography	Digital-to-silver hybrid systems, improved processing workflows	High-quality silver halides prints from digital images; archival and professional labs
2015–2024: Photography	Sustainable chemical practices, green gelatin alternatives, nanostructured silver halides	Environmentally friendly production, scientific imaging, nanoscale optical applications
1975–2025: Medicine/Biomedical	AgBr, AgCl emulsions or nanoparticles, photoactivated systems	X-ray films, antimicrobial coatings, photoactivated drug delivery
1975–2025: Cloud Seeding/Environment	AgI crystals, nanostructured ice nucleation agents	Precipitation enhancement, cloud seeding, improved ecological safety
1975–2025: Photocatalysis/Remediation	AgCl, AgBr, Ag/AgX composites, hybrid nanocatalysts	Degradation of dyes/pollutants, water treatment, enhanced photocatalytic efficiency
1975–2025: Electronics/Optoelectronics	Nano AgBr, AgCl, plasmonic and holographic systems	Holographic data storage, plasmonic sensors, nanoscale optical devices
2025: Emerging/Future Prospects	Ultra-fine nanostructured silver halides, hybrid quantum-photonic emulsions	Quantum sensing, ultrahigh-resolution imaging, advanced photonics, eco-friendly processes

**Table 7:** Integrated evidence of materials, chemical, and nanotechnology research leadership in interdisciplinary silver halides studies.

Category	Details (Publication Counts)	Implication for Discipline Leadership
Leading authors	Lo, Edward Chin Man (108); White, Allan H (67); Chu, Chun Hung (67)	These authors represent interdisciplinary leadership in silver halides research, integrating materials science, chemistry, physical chemistry, and nanotechnology, and guiding methodological and technological development in the field.
Leading contributing countries	China (18.3% of publications); United States (17.5%); India (8.7%); Germany (6.3%)	These countries provide research infrastructure, funding, and collaboration networks that drive silver halides science globally, establishing leadership in materials chemistry, photochemistry, and nanotechnology research.
Top institutions	Chinese Academy of Sciences (777 citations); Russian Academy of Sciences (647); CNRS, France (470)	Institutions serve as hubs for interdisciplinary silver halides research, innovation, and training, consolidating scientific authority and leadership in materials science, chemistry, and nanotechnology.
Primary journals of publication	Catalysis Today; Applied Catalysis B: Environmental; Journal of Materials Chemistry; Nature Materials; Langmuir; Organic Letters; Journal of Organic Chemistry; Chemical Reviews; Angewandte Chemie International Edition; Journal of the American Chemical Society	These journals function as central platforms for disseminating silver halides research, integrating materials science, chemistry, physics, nanotechnology, and environmental applications, and shaping discipline standards and collaboration.

*Note: Publications/citations clearly standardized as: total WoS citation count from affiliated publications*.

## Data Availability

No new data were created or analyzed in this study. Data sharing is not applicable to this article.
